# Carbon Tube-Based Cathode for Li-CO_2_ Batteries: A Review

**DOI:** 10.3390/nano12122063

**Published:** 2022-06-15

**Authors:** Deyu Mao, Zirui He, Wanni Lu, Qiancheng Zhu

**Affiliations:** 1 School of Mechanical and Automotive Engineering, Guangxi University of Science and Technology, Liuzhou 545006, China; maodeyu2012@126.com (D.M.); hzr13865666819@163.com (Z.H.); wannilu@126.com (W.L.); 2 Faurecia (Liuzhou) Automotive Interior Systems Co., Ltd., Liuzhou 545000, China

**Keywords:** carbon tube-based cathode, Li–CO_2_ battery, reaction mechanism, performance improvement, carbon neutrality

## Abstract

Metal–air batteries are considered the research, development, and application direction of electrochemical devices in the future because of their high theoretical energy density. Among them, lithium–carbon dioxide (Li–CO_2_) batteries can capture, fix, and transform the greenhouse gas carbon dioxide while storing energy efficiently, which is an effective technique to achieve “carbon neutrality”. However, the current research on this battery system is still in the initial stage, the selection of key materials such as electrodes and electrolytes still need to be optimized, and the actual reaction path needs to be studied. Carbon tube-based composites have been widely used in this energy storage system due to their excellent electrical conductivity and ability to construct unique spatial structures containing various catalyst loads. In this review, the basic principle of Li–CO_2_ batteries and the research progress of carbon tube-based composite cathode materials were introduced, the preparation and evaluation strategies together with the existing problems were described, and the future development direction of carbon tube-based materials in Li–CO_2_ batteries was proposed.

## 1. Introduction

Global warming caused by greenhouse gases is an essential factor affecting the current environmental deterioration. The generation of greenhouse gases is inevitable based on the biomass (coal, oil, natural gas, etc.) combustion energy conversion method. Among them, carbon dioxide is the most important greenhouse gas, standing as the core issue that needs to be addressed to realize the currently advocated “low carbon environment” [1]. The solution strategy from “carbon peak” to “carbon neutralization” is divided into two aspects: one is the need to reduce carbon emissions, that is, to minimize energy storage and transformation based on “C”, and to gradually promote all types of renewable alternative energy in all walks of life; the second is the treatment of existing carbon dioxide with conventionally applied methods including chemical conversion, photocatalytic reduction, electrochemical reduction, and biological conversion [2]. The conversion efficiency of these methods has yet to be improved, while the biggest limitation is that the direct conversion of C in CO_2_ inevitably requires additional energy (resulting in extra “carbon emissions”), as C in CO_2_ is in the highest oxidation state [3]. The products obtained by these methods are carbon monoxide, methane, ethylene, formic acid, methanol, etc. The gaseous or liquid products are involved in compression, packaging, storage, transportation, and other steps before they are used as energy storage materials, which is bound to cause further energy loss [4]. Thus, the conventional CO_2_ conversion method is also a “high carbon” process.

**Figure 1:**
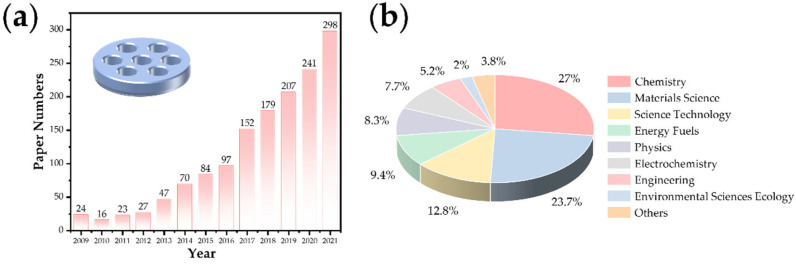
(**a**) The research progress and (**b**) distribution of disciplines of Li–CO_2_ batteries.

In recent years, the research on lithium–air batteries has made significant progress, especially the developments focusing on the optimal catalyst selection and the structure of the carbon matrix composite cathode design [5,6,7,8]. Because of their high theoretical specific capacity (their theoretical specific capacity is 5–10 times that of lithium-ion batteries) [5,9,10], they are considered the ultimate devices for the energy storage of vehicle power batteries in the future. In the study of lithium–oxygen batteries, the effects of water vapor and CO_2_ have to be carefully considered. Research shows that the battery capacity under an O_2_/CO_2_ mixture is three times that of pure oxygen [11], but the stability decreases significantly. The study of the battery reaction process in a carbon dioxide atmosphere is an essential intermediate link to realize the real application of metal–air batteries in the future [6,12]. With the expansion of this research, Li–CO_2_ batteries have gradually developed into an independent research direction because this system can achieve potential applications in particular fields such as Mars (96% of carbon dioxide in the atmosphere with a low temperature) detection [13] and energy storage for submarines. In recent years, the number of related research papers published (Web of Science statistics) has increased year by year (Figure 1a), and the distribution of disciplines is shown in Figure 1b. Previous studies have shown that lithium–carbon dioxide batteries based on carbon-based cathode catalysis can achieve a stable cycle, and it is believed that the charging and discharging process is based on the following reaction: 4Li^+^ + 3CO_2_ + 4e^−^→2Li_2_CO_3_ + C (E^0^ = 2.80 V versus Li/Li^+^) [14,15,16]. This reaction has attracted wide attention in the fields of energy and the environment because it involves the fixation and transformation of CO_2_ in the electrochemical energy storage process. With the deepening of this research, the reversibility of the battery reaction has also sparked a controversial concept. At present, significant progress has been made in the study of the performance improvement of battery systems, such as the number of rechargeable cycles and the reduction in the overpotential. However, the research on the controllable preparation of optimized electrode materials and the corresponding reaction mechanism is still in its infancy.

This review introduces the primary mechanism of lithium–carbon dioxide batteries and the latest progress in the application of carbon tube-based materials in battery systems, including the strategy and application of carbon nanotubes (fibers) combined with noble metals, molybdenum-based materials, other metal-based materials, and heteroatoms. This paper focused on the optimum selection and structure construction of carbon tube matrix composites and the improvement and enhancement of the performance of lithium–carbon dioxide batteries. Combined with innovative research methods, the development direction of carbon tube-based Li–CO_2_ batteries was proposed.

## 2. Structure and Reaction Mechanism of a Lithium–Carbon Dioxide Battery

### 2.1. The Structure of a Li–CO_2_ Battery

**Figure 2:**
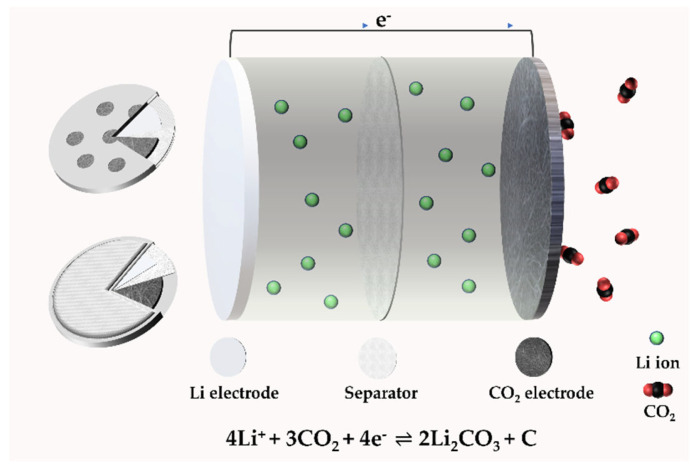
The structure of a Li–CO_2_ battery.

A typical type of Li–CO_2_ battery consists of a porous cathode, electrolyte (liquid, solid), and lithium metal anode [17]. The basic structure is shown in Figure 2, in the form of coin cells from different points of view.

### 2.2. The Mechanism of a Li–CO_2_ Battery

**Figure 3:**
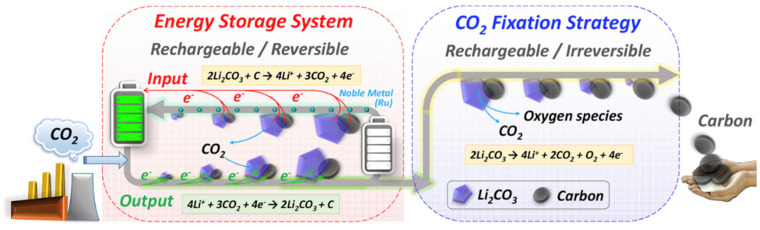
Schematic for the achievement of an energy storage system (reversible process) and a CO_2_ fixation strategy (irreversible process) via Li–CO_2_ electrochemistry technology. (Reprinted/adapted with permission from [18]. Copyright 2017 Elsevier).

The reaction process of Li–CO_2_ batteries is closely related to the electrode, electrolyte, and atmosphere environment. Studies have shown that lithium–carbon dioxide batteries cannot discharge in a pure CO_2_ atmosphere, and there must be a small amount of oxygen involved in the catalysis [11]. A much more critical problem is the lack of research evidence on the generation and decomposition process of discharge product C, and the lack of direct and powerful characterization test data. Zhou’s group reported in Joule that lithium–carbon dioxide batteries are rechargeable. Still, their charge and discharge processes are irreversible: lithium carbonate generated during discharge can be decomposed, but the generated carbon will not be decomposed but enriched on the electrode [18]. That is, when charging, the battery reaction is 2Li_2_CO_3_→2CO_2_ + O_2_ + 4Li^+^ + 4e^−^(E^0^ = 3.82 V versus Li/Li^+^). Based on this principle, an energy storage device can be designed, which can not only reduce the emission of CO_2_ but also use CO_2_ as the energy storage carrier. The gaseous CO_2_ is fixed into the solid C; that is, during the charging and discharging process, high specific energy storage and greenhouse gas treatment can be realized at the same time. The energy efficiency reaches 73.3% without pollution, which is of great significance to the solution of energy and environmental problems (Figure 3). Therefore, it is urgent to carry out proper mechanism research to confirm the reaction process of the system [19,20].

### 2.3. The Application of DEMS in Electrode Interface Reaction

**Figure 4:**
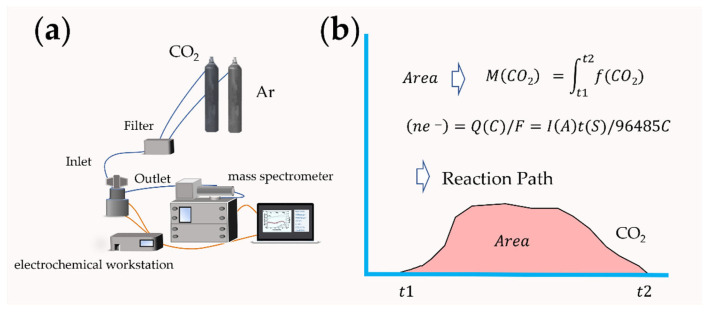
(**a**) Schematic for a DEMS system; (**b**) the process of predicting the reaction mechanism.

The macroscopic property of the electrode catalytic material interface lies in the chemical reaction. Since metal–air batteries involve the gas consumption and emission at the surface interface of catalytic materials, in recent years, the application of differential electrochemical mass spectrometry (DEMS) analysis based on gas detection in the field of lithium–oxygen batteries have realized the continuous measurement of gas, and in situ online analysis of the catalytic cathode interface reaction and possible side reactions [21,22]. The components of DEMS are shown in Figure 4a. In 2006, Bruce et al. added Li_2_O_2_ to the cathode of a lithium–air battery [23]. With the help of DEMS analysis, the results proved that O_2_ could be generated by oxidation during the charging process. The reaction mechanism of lithium–oxygen batteries was directly confirmed by experimental data. McCloskey et al. studied the formation and decomposition process of Li_2_O_2_ [21]. Peng et al. used nanoporous gold as a simulated cathode instead of a carbon-based cathode material [24]. The battery test results based on the DMSO electrolyte showed that no CO_2_ was detected. Based on the test of carbon-based materials, there will be apparent CO_2_ emission, indicating that lithium–air batteries with a carbon-based cathode have the possibility of decomposition in the use process, and the safety factor must be considered. The effect of nanocatalysts added to the cathode materials can also be evaluated by the DEMS test and the reaction measurement calculation, such as TiC and Mo_2_C, so as to speculate on the actual effect of the catalyst and analyze the stability of the electrode material [25]. The charge–discharge e^−^/reaction gas ratio can be calculated by Faraday’s law so as to speculate on the reaction path (Figure 4b). Another advantage of mass spectrometry is that it can be combined with isotope calibration methods, such as an isotope reaction gas or electrolyte, to track the intermediate products in the reaction process, and to effectively analyze the ion migration process and catalytic mechanism of the interface by in situ chemical testing methods [26].

## 3. Carbon Tube-Based Cathode for Li–CO_2_ Battery

Carbon nanotubes are unique 1D materials, consisting of hexagonal carbon atoms to form a single layer to dozens of layers of coaxial circular tubes. With special mechanical, thermal, and electrical properties, they can be used in special applications in the field of engineering materials. Carbon nanotubes have good electronic conductivity and form a unique 3D overlapping space structure, providing sufficient space for the deposition of discharge products [27]. In 2015, Zhou’s group applied carbon nanotubes to a Li–CO_2_ battery cathode, and the battery charge and discharge cycles were realized [28]. However, the catalytic activity of pure carbon materials in the charge–discharge reaction of the Li–CO_2_ battery was limited, and the battery exhibited a high charge–discharge overpotential and a poor cycle life. Carbon tube matrix composites can effectively encapsulate and load various catalyst nanoparticles so as to achieve the expected carbon dioxide reduction/evolution reaction (CO_2_-RR/CO_2_-ER) catalytic performance and increase the reactive sites in Li–CO_2_ batteries.

### 3.1. Carbon Tube–Noble Metal-Based Composites

Noble metals such as gold, silver, and the platinum family (ruthenium, rhodium, palladium, osmium, iridium, platinum) have unique activity in catalytic reactions [29,30]. They are used as catalysts for oxygen reduction (ORR) and oxygen precipitation (OER) reactions in the initial stage of lithium–air battery research and are designed as air electrodes with various unique spatial structures [31].

Kong et al. dispersed Au nanoparticles uniformly on the surface of a carbon tube (AuNPs/CNTs) to achieve 46 cycles of a Li–CO_2_ battery under the condition of a limited capacity of 100 mAh g^−1^ [32]. In the discharge stage, the voltage platform is obviously higher than that of the carbon nanotube electrode, indicating that it has a catalytic effect in the CO_2_ reduction process. In the charging stage, the voltage platform and carbon nanotube electrode are basically the same, reaching more than 4.5 V, indicating that the catalyst effect of the Au electrode in the CO_2_ evolution process is limited.

**Figure 5:**
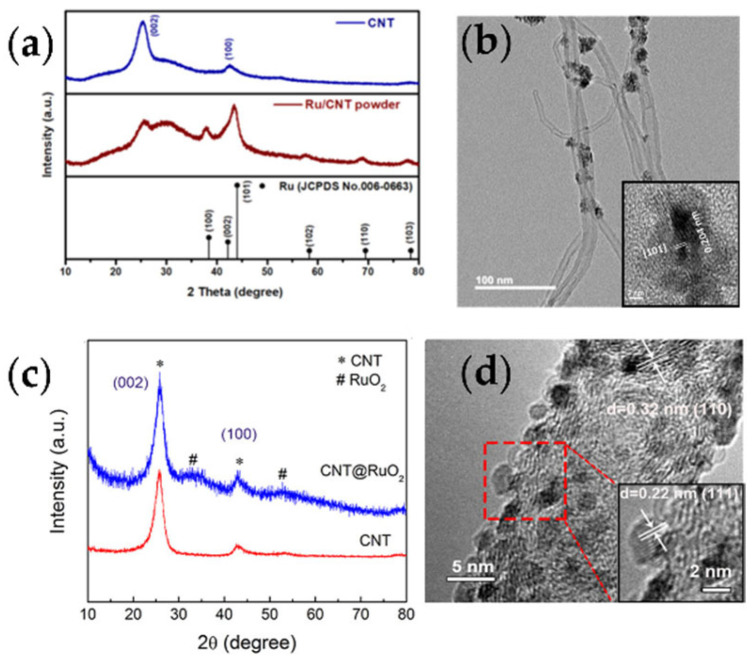
(**a**) XRD patterns and (**b**) TEM and high-resolution TEM images of Ru/CNT (reprinted/adapted with permission from [36]; Copyright 2021 American Chemical Society); (**c**) XRD patterns and (**d**) HRTEM image of CNT@RuO_2_ (reprinted/adapted with permission from [38]; Copyright 2019 American Chemical Society).

In previous work, ruthenium-based materials were considered efficient catalysts for lithium carbonate decomposition [33,34]. Chen et al. anchored Ru nanoparticles on carbon tubes, which could effectively improve the conductivity of the material matrix, and the porous skeleton formed by cross-linking could promote the diffusion and transmission of CO_2_ and the electrolyte [35]. By comparing the attenuation of the battery system at a low specific capacity of 100 mAh g^−1^ and a high specific capacity of 500 mAh g^−1^, the researchers believed that the dominant role is not the passivation of the cathode, but the effect of lithium dendrites on the anode. After replacing the lithium metal sheet with a C/Li anode deposited on the surface by sputtering, the cycle stability of the battery can be improved by more than three times. Using ruthenium chloride and carbon tubes as raw materials, ruthenium nanoparticles can be attached to the surface of carbon tubes by the reflux method (Figure 5a,b). The obtained composite material can be used as the cathode of lithium–carbon dioxide batteries and sodium–carbon dioxide batteries to realize more than 100 cycles [36]. Li et al. combined a covalent organic framework compound (COF) with Ru-coated carbon nanotubes to explore the adsorption capacity of the material for CO_2_ [37]. In the Li–CO_2_ battery, the specific capacity reached 27348 mAh g^−1^. The good performance was attributed to the 1D channel of the material and the functionality of the COF, which facilitate the capture of CO_2_ and the rapid transmission of lithium ions. These synergistic effects promote the rapid formation/decomposition of Li_2_CO_3_ during the discharge–charge process.

Using ruthenium chloride and carbon nanotubes as raw materials, through a simple solution reaction and vacuum sintering at 200 °C, the structure of RuO_2_ particles attached to the surface of carbon nanotubes can be obtained (Figure 5c,d) [38]. This type of material was first used in Li–CO_2_ batteries. Under the condition of a limited specific capacity of 500 mAh g^−1^, the initial cycle was 30 cycles, and the charging voltage was lower than 4.0 V. Based on the analysis of XPS, XRD, and SEM, most of the discharge products of lithium carbonate can be effectively decomposed. The catalytic activity of the electrode was analyzed by charging decomposition with pre-filled lithium carbonate. The coulombic efficiency of the carbon tube electrode was 56%, and the charging voltage was close to 4.5 V. The coulombic efficiency of the carbon tube and ruthenium oxide composite electrode was 93%, and the charging voltage was controlled at 3.9 V.

In the practical application of noble metal-based electrode materials, the following issues should be considered: raw material cost, synthesis technology, and performance optimization. Alloying is an effective strategy to reduce costs and optimize the composition. Jin et al. attached Ru and Cu nanoalloys on the surface of carbon nanofibers. The optimized composition and electronic effect can promote the formation and decomposition of lithium carbonate during discharging and charging [39].

### 3.2. Carbon Tube–Molybdenum-Based Composites

Mo-based materials, due to the multiple valence states of Mo, its ease in the formation of composites, its high electronic conductivity, its high electrocatalytic activity, and the low cost of raw materials, have achieved excellent electrochemical performance in the study of Li-O_2_ batteries [40,41]. In Li–CO_2_ batteries, Mo-based materials also exhibited excellent properties, such as: improved catalytic activity, reduced charging overpotential, and long cycle stability [42].

**Figure 6:**
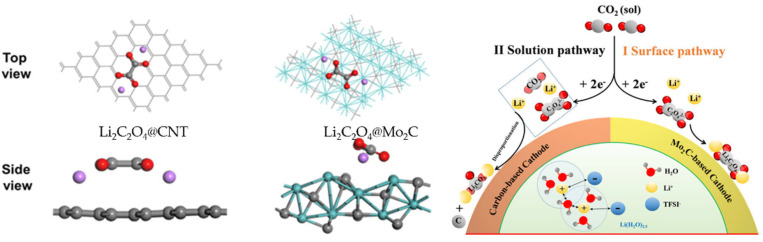
Schematic of the electrochemistry mechanism of CO_2_ reduction in WIS-based Li–CO_2_ batteries with various cathodes (reprinted/adapted with permission from [45]; Copyright 2021 American Chemical Society).

Molybdenum carbide (Mo_2_C) has been widely studied due to its excellent catalytic properties, similar to VIII metals. It has attracted extensive attention in methane reconstruction, water–gas transfer reactions, hydrogen evolution reactions, and CO_2_ reduction reactions. Compared with the metal Mo, the high activity of Mo_2_C originates from the electronic properties introduced by carbon, which affects the reaction activity of the Mo-C bond energy and adsorbate. Chen’s research group synthesized a composite of Mo_2_C and carbon nanotubes and used it for the test of Li–CO_2_ batteries [43]. The battery energy efficiency reached 77% and could be recycled for 40 cycles. The mechanism analysis showed that Mo_2_C can stabilize the intermediate product of CO_2_ in the reduction process during discharging, thereby inhibiting the formation of insulating lithium carbonate. The amorphous discharge product Li_2_C_2_O_4_-Mo_2_C can be decomposed at a 3.5 V charge voltage. First-principles calculation can further analyze the role of Mo_2_C electrodes in Li–CO_2_ batteries. Yang et al. systematically studied the Gibbs free energy changes of Li_2_C_2_O_4_ and Li_2_CO_3_ nucleation intermediates and theoretically proved that Li_2_C_2_O_4_ can stably become the final discharge product without forming Li_2_CO_3_ [44]. The overpotential was analyzed by an electrochemical free-energy level diagram, and the catalytic activity of the catalyst during charging and discharging was evaluated. The electron transfer between the intermediate product and the Mo_2_C catalyst plays a crucial role in the stability of the discharge product and the electrochemical mechanism of the battery. Xia’s group designed a water-in-salt electrolyte (LiTFSI/H_2_O 21.0 mol/1 kg) and studied the reaction mechanism of a Li–CO_2_ battery composed of CNT and Mo_2_C/CNT cathodes [45]. Through a variety of in situ/non-in situ and qualitative/quantitative characterization analyses, the electrode based on Mo_2_C/CNT can realize the reversible conversion between CO_2_ and Li_2_C_2_O_4_ at a low charge–discharge overpotential. In contrast, the CNT-based electrode needs to form and decompose Li_2_CO_3_ at a high overpotential. The electrochemistry mechanism is schematically shown in Figure 6.

MoC has also been demonstrated as a highly efficient catalyst in lithium–air batteries [46]. Zhu et al. applied a modified flowing catalyst chemical vapor deposition (FCCVD) method by using dicyandiamide as a carbon and nitrogen source [47]. Nitrogen-doped carbon nanotubes were grown on the surface of the nickel foam skeleton, and MoC nanoparticles were embedded in the carbon nanotubes. The obtained self-supporting structural materials can be directly used for Li–O_2_ batteries, Li–CO_2_ batteries, and Li–air batteries to avoid the addition of an organic binder. The formation and decomposition of the discharge product lithium carbonate on the electrode surface were verified by XRD, Raman, XPS, FTIR, and SEM analysis. Combined with the DEMS test, the possible reaction mechanism in the charging and discharging process was explained.

Chen et al. prepared an array structure of MoO_3_-coated CNTs grown on the surface of a nickel foam [48]. The cross-linking structure of carbon tubes provided abundant channels for electron transport. The large specific surface area provided sufficient ion embedding/desorption sites. Based on the surface self-restriction and self-saturation adsorption, the MoO_3_ layer deposited on the outer layer of the carbon tube had good 3D consistency. The Li–CO_2_ battery assembled with the material had a discharge capacity of 121.06 mAh cm^−2^, a charge voltage of less than 3.8 V, and a cycle number of 300 cycles.

Transition metal dichalcogenides have been widely investigated in various electrochemical reactions, including Li–CO_2_ batteries [34,49]. The composites of MoS_2_ and CNTs also realized a stable cycle in Li–CO_2_ batteries [50]. Chen et al. prepared a MoS_2_/CNT electrode with a discharge capacity of 8551 mAh g^−1^, coulombic efficiency of 96.7% and charge voltage of 3.98 V. The DFT calculation results showing the adsorption sites of Li, CO_2_, and Li_2_CO_3_ on MoS_2_ materials were analyzed and verified by Raman and X-ray absorption fine structure spectra. In addition, MoSe_2_@CNT composites were designed, synthesized, and proven to be beneficial to the nucleation and growth of Li_2_O_2_ in Li–O_2_ batteries [51]. The batteries had a specific capacity of 32,000 mAh g^−1^ and a cycle life of 270 at 500 mA g^−1^.

### 3.3. Carbon Tube–Other Metal-Based Composites

Transition metals are widely used in various catalytic fields due to their rich con-tent, low cost, and good catalytic activity. Copper was demonstrated effective in the adsorption and activation of CO_2_ molecules [52,53,54]. Xu et al. prepared copper polyphthalocyanine-carbon nanotubes composites (CuPPc-CNTs) by solvothermal in-situ polymerization method. The obtained Li–CO_2_ battery show high discharge capacity [55].

Li et al. used an FCCVD method to grow nitrogen-doped carbon tubes on a Ti substrate [56]. The electrode with a self-supporting structure had 45 cycles in a Li–CO_2_ battery. The electrode could also assemble a flexible fibrous battery with a semi-solid electrolyte.

Kim et al. used hemoglobin biomolecules as raw materials to analyze the mechanism of Fe nanoparticles embedded in nitrogen-doped carbon nanotubes through capillary action under different heat treatment conditions [57]. Non-in situ characterization proved that the material was not Li_2_C_2_O_4_ based on the formation and decomposition of Li_2_CO_3_ during the battery cycle. It is believed that the material has high catalytic activity in the battery reaction due to the formation of an Fe-O-C bond.

Thoka et al. compared the performance of CNTs, Co_3_O_4_@CNT and ZnCo_2_O_4_@CNT in Li–CO_2_ batteries [58]. The ZnCo_2_O_4_@CNT composite with a spinel structure significantly decreased the overpotential while improving the cycle number performance of the battery.

Zhang et al. deposited NiO nanosheets on the CNT surface using a hydrothermal method. In the first full charge–discharge cycle, the coulombic efficiency reached 97.8%, and the efficiency after five cycles was 91.7% [59]. Inspired by the design of high-nickel and low-cobalt electrode materials, Xiao et al. modified Co_0.1_Ni_0.9_O_x_ nanoparticles on the surface of carbon nanotubes [60]. Through various characterization techniques, the effect of doping Co on the electrochemical performance was systematically studied. The Co_0.1_Ni_0.9_O_x_/CNT electrode achieved 50 cycles without apparent attenuation, which is twice more than that of the NiO/CNT and CNT electrodes. The improvement of the catalytic performance was attributed to the advancement in the P-type electronic conductivity by doping Co^2+^ in the NiO lattice. The Co^2+^-doped electrode surface formed polymer-like discharge products, which was considered to build a better reaction interface.

Lei et al. prepared an α-MnO_2_/CNT electrode, which could increase the number of surface-active sites. A total of 50 cycles could be realized under the limited capacity, and 6 cycles could be realized under the full charge and discharge conditions [61]. Liu et al. prepared layered sodium manganese hydroxide δ-MnO_2_-coated carbon nanotubes [62]. Porous lamellar MnO_2_ had abundant oxygen vacancy and was evenly distributed on the surface of the carbon tubes. The 3D cross-linked pore network structure formed between the layered MnO_2_ and CNTs could effectively promote the permeation of the electrode solution and the diffusion and transmission of CO_2_, providing a sufficient Li^+^/electron transport path and catalytic activity sites for the CO_2_ precipitation reaction. The adsorption energies of CO_2_ and Li_2_CO_3_ in the absence and presence of oxygen vacancies were calculated by DFT, indicating that CO_2_ is easier to adsorb at sites containing oxygen vacancies.

**Figure 7:**
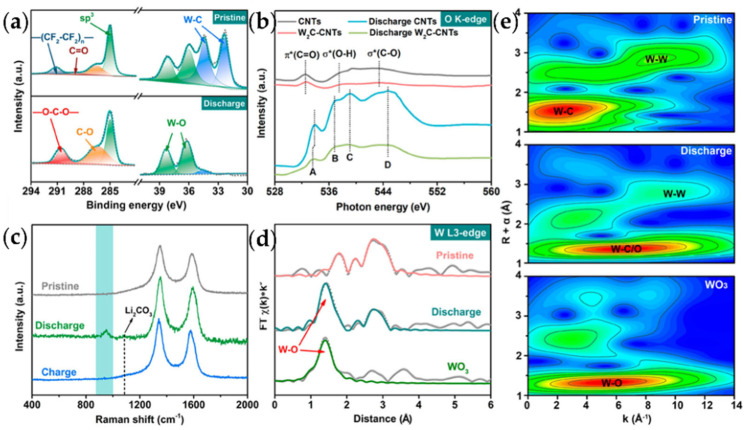
Investigation of the reaction mechanism of Li–CO_2_ batteries with W_2_C-CNTs. (**a**) XPS spectra, (**b**) O K-edge XANES spectra, (**c**) Raman spectra, (**d**) wavelet transform EXAFS spectra, and (**e**) Fourier transform EXAFS spectra of the cathode (reprinted/adapted with permission from [63]; Copyright 2021 American Chemical Society).

Zhang et al. embedded tungsten carbide (W_2_C) nanoparticles into the CNT wall to achieve an ultra-low charging voltage of 3.2 V in lithium–carbon dioxide batteries [63]. The ultra-low polarization originates from the electron-rich effect of W atoms in the W-O bond, breaking the stable triangular structure in CO_3_^2-^. The discharge product formed by the W_2_C-CNT catalytic electrode was amorphous, which is conducive to decomposition at a low voltage, and the cycle energy efficiency of the battery could reach 90.1%. Combined with the EXAFS spectrum and theoretical calculation results, it was considered that the interaction between lithium carbonate and CNTs was physical, while the interaction between lithium carbonate and W_2_C-CNTs was chemical (Figure 7). The problem with the electrode material is that the charging voltage began to increase significantly after 30 cycles.

### 3.4. Heteroatom-Doped Carbon Tube-Based Composites

**Figure 8:**
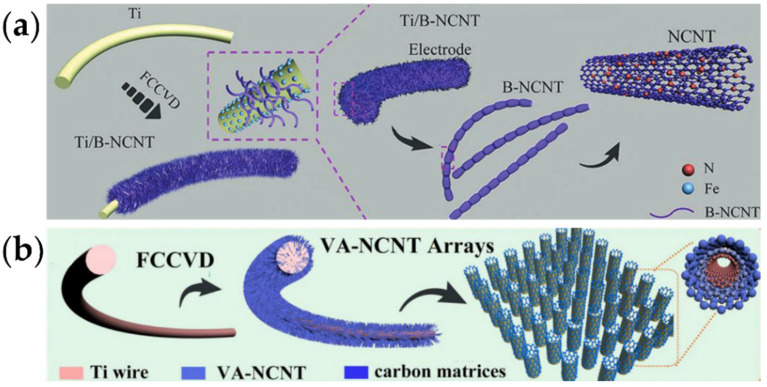
(**a**) Schematic illustration of preparation procedures for B-NCNT electrodes using an FCCVD method (reprinted/adapted with permission from [66]; Copyright 2019 Wiley); (**b**) schematic illustration of the synthesis procedure of VA-NCNT arrays on a Ti wire via an FCCVD method (reprinted/adapted with permission from [69]; Copyright 2020 American Chemical Society).

Due to their low catalytic activity, pure carbon tubes have been proven not suitable for direct use in lithium–carbon dioxide batteries, and a proper adsorbent should be designed [64,65]. Heteroatom doping is an effective strategy. For example, the introduction of nitrogen atoms can enhance the adsorption of CO_2_ on the surface through Lewis’s acid–base interaction [47]. Li et al. prepared bamboo-like nitrogen-doped carbon tubes using an FCCVD method (Figure 8a) [66]. Studies have shown that nitrogen-doped carbon tube materials can benefit from pyridine nitrogen on the surface, forming abundant defect active sites. Li et al. believed that carbon-based materials were not suitable for direct use in lithium–carbon dioxide batteries without binders, and the introduction of binders would not only cause the loss of active sites on the electrode but also lead to heterogeneous dispersion, resulting in attenuation of the catalytic activity [67,68]. In addition, the contact interface between the catalyst and lithium carbonate will also decrease, resulting in incomplete decomposition, and gradually accumulating and blocking the channels of CO_2_ and Li^+^ transport, thereby further reducing the active sites. High-capacity Li–CO_2_ batteries have become candidates for a flexible power supply, having potential applications in bracelets or wearable electronic devices. On this basis, a vertical array of nitrogen-doped carbon tubes on a titanium mesh was designed, which was used as a self-supporting electrode to achieve an ultra-long cycle, high performance and a flexible lithium–carbon dioxide battery with a gel polymer electrolyte. Two layers of nitrogen-doped graphene-coated CNTs were prepared by Dai’s group using anodic aluminum oxide (AAO) as a template (Figure 8b) [69]. They can also be used as a binder-free 3D cathode, as well as a metal-free structure. Song et al. prepared N-S co-doped carbon tubes and confirmed N-S co-doping by high-resolution XPS [70]. The battery test analyzed the high catalytic activity of the composite compared with the CNTs, and a specific capacity of 23,560 mAh g^−1^ could be achieved in the quasi-solid flexible lithium–carbon dioxide battery for 110 days, while the effect of N-S doping remains to be further studied.

In summary for this section, the current research progress of CNT-based cathodes for Li–CO_2_ batteries is compared in Table 1. The discharge capacity with the corresponding current density, the cycle performances, and the discharge–charge voltage platform are listed in detail.

## 4. Prospect

The actual process of a Li–CO_2_ or future genuine Li–air battery is complex [73]. Based on the existing structure and system, a large number of creative works have been carried out, including the battery reaction process assisted by an external light field, the replacement of a liquid electrolyte with a solid one, and other types of metal–carbon dioxide batteries.

### 4.1. Light Field Assistance

**Figure 9:**
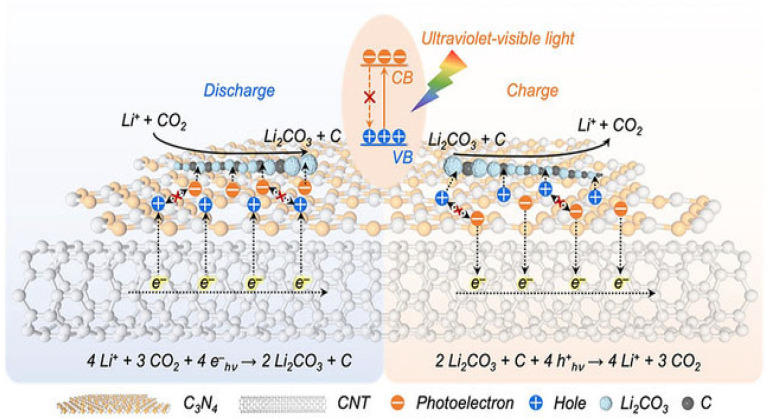
Depiction of the light-assisted discharge–charge processes in a CNT@C_3_N_4_ heterostructured photocathode (reprinted/adapted with permission from [74]; Copyright 2022 Wiley).

Peng’s group designed a carbon nanotube framework with an internal connection structure and coated carbon nitride (C_3_N_4_) on the surface to form a heterojunction photocathode for a quasi-solid-state Li–CO_2_ battery [74]. The composite was prepared at 600 °C to form carbon nitride with more defect structures, which enhanced the absorption of ultraviolet light and produced abundant photoelectrons and vacancies. This further led to the red shift of the absorption edge and the improvement of the carrier separation efficiency in carbon nitride. The accelerated transport of the charge between the carbon nitride and carbon tube structure provides more photoelectron/vacancy migration and promotes the reduction/evolution reaction of carbon dioxide rather than recombination. This process also increases the utilization of light energy. Carbon dioxide was reduced to lithium carbonate/carbon by photoelectrons at 3.24 V (higher than the thermodynamic equilibrium voltage, 2.80 V). In the charging process, the voltage of the oxidation process was only 3.28 V, so the energy efficiency of the charging and discharging cycle process was 98.8%, while the energy efficiency after 100 cycles was 86.1%. This electrode material can also adapt to the application of flexible wearable electronic equipment in the future. Based on the photoelectric effect, the specific incident light can excite a large number of high-energy photoelectrons and holes with high redox activity on semiconductor materials, which is expected to promote the reduction/evolution of CO_2_ (Figure 9). However, due to the unfavorable charge transfer/separation of typical semiconductor photocatalysts, most photogenerated carriers often recombine before participating in the target battery reaction. The charge transfer/separation of the semiconductor photocatalyst is unfavorable, and most photogenerated carriers tend to recombine before participating in the target battery reaction. Only a tiny fraction of photogenerated carriers can migrate to the surface of semiconductors to promote the CO_2_ reduction/evolution reaction. In order to effectively use light to promote the kinetic process of a CO_2_ cathode, it is necessary but challenging to encourage the transfer/separation of photogenerated carriers in a semiconductor photocatalyst. Peng’s team designed a synergistic two-field auxiliary cathode to solve the inherent limitation of semiconductor photocatalysts by combining plasma metal nanoparticles (such as gold and silver) with semiconductor photocatalytic materials [75]. Under the incident light, the free electrons in the plasma metal nanoparticles can construct a locally enhanced electric field, exerting the opposite force on the electrons and holes to suppress the recombination of carriers. Silver (Ag) nanoparticles were electrodeposited on an anodic oxidation TiO_2_ nanotube array as a dual-field auxiliary cathode to promote the CO_2_ reduction/evolution reaction. Under the action of a light field, a large number of photogenerated electron holes are generated on TiO_2_, and the enhanced electric field around Ag nanoparticles promotes the separation/transfer of photogenerated carriers, thus making better use of carriers for the CO_2_ reduction/evolution reaction. The dual-field-assisted Li–CO_2_ battery had an ultra-low charging voltage (2.86 V at 0.10 mA cm^−2^), and the efficiency was 86.9% after 100 cycles, achieving an area capacity of 31.11 mAh cm^−2^.

### 4.2. Solid State

In recent years, the research on Li–CO_2_ batteries has been mainly based on the non-proton solvent system, namely, the organic electrolyte system [76,77]. Compared with carbonate electrolytes used in lithium-ion batteries, ether or sulfone electrolytes have wider electrochemical windows and better stability. However, with lithium metal as a negative electrode, it is inevitable that the problems of lithium dendrite growth and a short circuit caused by the penetrating diaphragm will be faced [78,79]. At the same time, as an open system, the battery has problems such as volatilization, leakage of liquid electrolytes, and dissolution of the atmosphere in the air into the corrosion of the lithium sheet, which seriously restrict the improvement of battery performance in terms of stability and a long cycle life. Solid electrolytes have the following advantages:

1. They avoid electrolyte volatilization, which is not flammable;
2. They can inhibit the growth of lithium dendrites with higher safety;
3. They are not prone to inducing side reactions and have a better stability;
4. They can effectively prevent water vapor in the air and reduce the corrosion of lithium anodes.

However, there are still many bottlenecks to be considered in the development and application of solid-state Li–CO_2_ batteries, including the composition and structure design of the electrolyte, the contact between the electrolyte and electrode solid interface, the ionic and electronic conductivity and the cost [80]. In existing solid electrolytes, sulfur-based and garnet-based electrolytes are sensitive to water vapor and need to be prepared in an inert atmosphere, which is not suitable for metal–air batteries. Sodium-ion fast-charged Li_1+x_Al_x_Ge_2-x_(PO_4_)_3_ (LAGP) and Li_1+x_Al_x_Ti_2-x_(PO_4_)_3_ (LATP) ceramics can alleviate the grain-limited boundary transport of lithium ions and stabilize in a carbon dioxide atmosphere [81]. Using a commercial LAGP wafer as the electrolyte, a Ru/CNT cathode and a lithium anode, Liu et al. assembled a Li–CO_2_ battery and achieved 45 cycles at a limited capacity of 500 mAh g^−1^ [31].

Wang’s group prepared a gel polymer electrolyte with a compact structure and good thermal stability by conventional UV curing technology and assembled rechargeable Li–CO_2_ batteries based on carbon tube gas electrodes [82]. Under the condition of a limited specific capacity of 1000 mAh g^−1^, the battery could cycle for 60 cycles. Compared with the liquid electrolyte battery, the discharge product had a granular form with poor crystallinity. In the liquid electrolyte battery, the discharge product created a continuous polymer coating structure, which is not conducive to the transmission of ions, electrons, and gases, thus affecting the cycle stability of the battery. The charging process of the battery was analyzed by in situ differential electrochemical mass spectrometry, but the reaction mechanism needs to be further verified by the isotope method.

**Figure 10:**
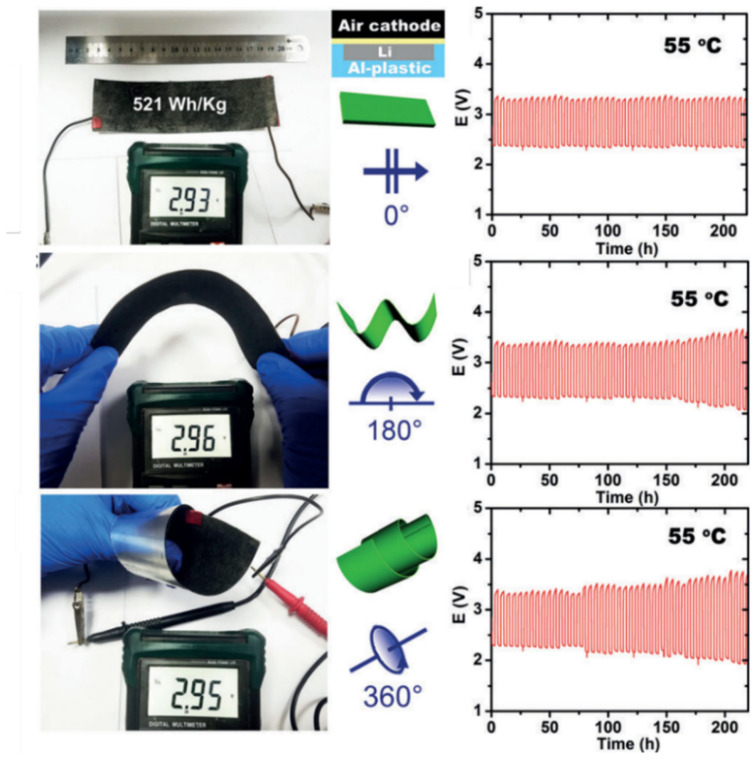
The bending and twisting properties and corresponding cycling numbers of Li–CO_2_ batteries (reprinted/adapted with permission from [83]; Copyright 2017 Wiley).

The selection of both the electrode and electrolyte needs to be considered in flexible batteries [67]. Li–CO_2_ batteries with a liquid electrolyte inevitably have a leakage problem, which cannot meet the requirements of flexible batteries with high safety. Based on a lithium sheet anode and a polymethacrylate/polyethylene glycol-lithium perchlorate-silica composite polymer electrolyte combined with a carbon nanotube cathode, Chen’s team constructed a flexible Li–CO_2_ battery without a binder and liquid electrolyte [83]. At 55 °C, the ionic conductivity of this organic–inorganic composite electrolyte reached 0.0714 mS cm^−1^, while the composite of the electrode and electrolyte provided more surface-active sites, reduced the interfacial resistance, and avoided the side reactions caused by the binder. The assembled Li–CO_2_ battery effectively cycled for 100 cycles with a specific capacity of 1000 mAh g^−1^. The assembled soft-packed battery achieved a capacity of 993.3 mAh, reached an energy density of 521 Wh kg^−1^ and cycled for 220 h under the bending condition of 0–360° (Figure 10). The formation and reaction process of lithium carbonate were characterized by in situ Raman technology, and the stability of the composite electrolyte/electrode material was verified.

### 4.3. Other Metal–CO_2_ Batteries

Due to the shortage of lithium metal resources and the complicated and expensive production process, it has become a hot trend to derive metal–carbon dioxide batteries from other metals (such as sodium or potassium as compared in Table 2) with a higher content in the Earth’s crust. Sodium is similar to lithium in terms of physical and chemical properties. The theoretical energy density of sodium–carbon dioxide batteries is 1013 Wh kg^−1^, and the reaction is based on 4Na + 3CO_2_→2Na_2_CO_3_ + C [84,85,86,87]. The discharge voltage of this type of battery is 2.35 V, which is lower than that of a Li–CO_2_ battery. Still, the advantage is that the charging voltage is reduced accordingly, which has better safety [36]. Because metal sodium is more active and the atomic radius is larger, it will cause higher polarization and more security problems. At present, sodium–carbon dioxide (Na–CO_2_) primary batteries and rechargeable batteries have great security risks, mainly due to the problems with the liquid electrolyte and pure sodium anode, including the leakage and volatilization of the liquid electrolyte in sodium salt/organic solvent Na–CO_2_ batteries (especially under high-temperature working conditions). During the battery cycle, dendrite formation or surface cracking will also occur, leading to a short circuit. Therefore, it is crucial to find alternatives to liquid electrolytes and pure sodium anodes [88]. In potassium–carbon dioxide (K–CO_2_) batteries, using an alloy instead of a pure metal anode will be safer and more stable and will avoid the generation of dendrites, which is an effective strategy. A K–CO_2_ battery was assembled with potassium-tin alloy as the negative electrode and carbon tubes containing carboxyl functional groups as the positive electrode. Since the -COO^−^ ions in the carboxyl group were similar to those in potassium carbonate, the C=O bond in potassium carbonate could be weakened, thus promoting the decomposition of potassium carbonate [89]. The specific capacity of the battery reached 3681 mAh g^−1^ at the current density of 100 mA g^−1^; under the condition of a limited capacity, 400 cycles could be realized.

## 5. Conclusions

This review introduced the basic structure and reaction mechanism of Li–CO_2_ batteries, focusing on the application of carbon nanotube-based composites in Li–CO_2_ batteries, from material synthesis to performance analysis. New development directions of optical field assistance, the solid state, and other metal–CO_2_ batteries were also introduced as prospects. Although many breakthroughs have been made, Li–CO_2_ batteries still have many problems to be solved, including the following:

- Mechanism problems in actual material systems. Through mass spectrometry or other in situ characterization techniques, the dynamic process of the battery system is usually explored by using simulated batteries, which may not effectively correspond to the actual battery system, especially in terms of the battery performance under different specific electrode material systems. For several steps of intermediate product formation, lithium carbonate or Li_2_C_2_O_4_ needs to be combined with the specific actual material system, and even with the different phase structures and tri-configuration systems of the same material, which is complex work.
- Key material selection and structural design issues. The selection of catalytic electrode materials needs to comprehensively consider factors such as the source, cost, preparation process, catalytic performance, and stability. The selection of the electrolyte is primarily concerned with the safety of the voltage window range, as well as the cost. The cost of existing liquid and solid electrolyte systems is much higher than that of lithium-ion batteries. In addition to the electrode and electrolyte, the type and packaging process of the separator and battery casing will affect the final performance of the battery. That means each component plays a vital role in the final performance and application of Li–CO_2_ batteries.
- Characterization method. Non-in situ characterization methods can only provide complementary reference information for the disassembled battery. Effectively combining in situ infrared, Raman, scanning, and transmission electron microscopy with the battery system is the key to the study. In addition, with the combination of experimental characterization and theoretical calculation, the experimental data should provide more guidance to the calculation model, rather than a simple simulation, such as the reaction path, product type, formation, and decomposition of energy, reactant, product adsorption energy, Gibbs free energy change, electron, ion migration rate of thermodynamics, and kinetics analysis.
- Practical application problems. As a high-energy-density energy storage device and carbon dioxide treatment device, the actual reaction process, performance, and effect of the battery at the amplification scale should be considered in the actual application process.

To sum up, Li–CO_2_ batteries are a very promising energy storage device and have the functions of CO_2_ absorption, fixation, and conversion. They are an important technical means for assisting energy optimization and environmental protection to realize carbon neutralization. However, many scientific and technical problems still need to be studied and solved steadily.

## Figures and Tables

**Table 1:** Comparisons of the performances of CNT-based cathodes for Li–CO_2_ batteries.

Cathode	Discharge Capacity/ Current Density	Cycle Performance (Cutoff Specific Capacity/Current Density)	Discharge–Charge Voltage Platform	Year	Ref.
Mo_2_C/CNTs	1150 μAh/20 μA	40 (100 μAh/20 μA)	2.65/3.35 V	2017	[43]
MoC/N-CNTs	8227 mAh g^−1^/100 mA g^−1^	90 (1000 mAh g^−1^/1000 mA g^−1^)	2.75/3.79 V	2017	[47]
NiO-CNTs	9000 mAh g^−1^/100 mA g^−1^	42 (1000 mAh g^−1^/50 mA g^−1^)	2.75/4.00 V	2018	[59]
COF-Ru@CNT	27,348 mAh g^−1^/200 mA g^−1^	200 (1000 mAh g^−1^/1000 mA g^−1^)	2.53/4.27 V	2019	[37]
CNTs@RuO_2_	2187 mAh g^−1^/50 mA g^−1^	55 (500 mAh g^−1^/50 mA g^−1^)	2.48/3.90 V	2019	[38]
N-CNTs@Ti	9292.3 mAh g^−1^/50 mA g^−1^	45 (1000 mAh g^−1^/250 mA g^−1^)	2.60/4.18 V	2019	[56]
MnO_2_/CNTs	7134 mAh g^−1^/50 mA g^−1^	50 (1000 mAh g^−1^/100 mA g^−1^)	2.62/3.95 V	2019	[61]
N-CNTs	23,328 mAh g^−1^/50 mA g^−1^	360 (1000 mAh g^−1^/1000 mA g^−1^)	2.72/3.98 V	2019	[66]
Ru/CNTs	2882 mAh g^−1^/100 mA g^−1^	268 (100 mAh g^−1^/100 mA g^−1^)	2.56/4.01 V	2020	[35]
ZnCo_2_O_4_@CNTs	4275 mAh g^−1^/100 mA g^−1^	230 (500 mAh g^−1^/100 mA g^−1^)	2.52/4.22 V	2020	[58]
Co_3_O_4_@CNTs	2473 mAh g^−1^/100 mA g^−1^	43 (500 mAh g^−1^/100 mA g^−1^)	2.45/4.38 V	2020	[58]
3D NCNTs/G	17,534 mAh g^−1^/50 mA g^−1^	185 (1000 mAh g^−1^/100 mA g^−1^)	2.77/3.90 V	2020	[69]
N,S-CNTs	23,560 mAh g^−1^/200 mA g^−1^	538 (500 mAh g^−1^/200 mA g^−1^)	2.63/4.52 V	2020	[70]
Ru/CNTs	4541 mAh g^−1^/100 mA g^−1^	45 (500 mAh g^−1^/100 mA g^−1^)	2.76/4.24 V	2021	[31]
AuNPs/CNTs	6399 mAh g^−1^/100 mA g^−1^	46 (1000 mAh g^−1^/200 mA g^−1^)	2.73/4.30 V	2021	[32]
Ru/CNTs	23,102 mAh g^−1^/100 mA g^−1^	100 (500 mAh g^−1^/100 mA g^−1^)	2.60/4.09 V	2021	[36]
Mo_2_C/CNTs	0.5 mAh/0.05 mA	20 (1000 mAh g^−1^/100 mA g^−1^)	2.74/3.41 V	2021	[45]
MoO_3_@CNTs	30.25 mAh cm^−2^/0.05 mA cm^−2^	300 (1 mAh cm^−2^/0.05 mA cm^−2^)	2.68 /4.03 V	2021	[48]
MoS_2_/CNTs	8551 mAh g^−1^/100 mA g^−1^	140 (500 mAh g^−1^/100 mA g^−1^)	2.70/3.94 V	2021	[50]
Fe/CNTs	3898 mAh g^−1^/100 mA g^−1^	30 (600 mAh g^−1^/100 mA g^−1^)	2.62/4.24 V	2021	[57]
Co_0.1_Ni_0.9_O_x_/CNT	5871.4 mAh g^−1^/100 mA g^−1^	50 (500 mAh g^−1^/100 mA g^−1^)	2.55/3.94 V	2021	[60]
CNT@MnO_2_	-	50 (1000 mAh g^−1^/200 mA g^−1^)	2.64/4.19 V	2021	[62]
W_2_C-CNTs	10,632 mAh g^−1^/100 mA g^−1^	75 (500 mAh g^−1^/200 mA g^−1^)	2.81/3.20 V	2021	[63]
N-CNTs	18,652 mAh g^−1^/100 mA g^−1^	120 (1000 mAh g^−1^/250 mA g^−1^)	2.51/4.25 V	2021	[67]
CuPPc-CNTs	18,652.7 mAh g^−1^/100 mA g^−1^	160 (1000 mAh g^−1^/200 mA g^−1^)	2.87/4.32 V	2022	[55]
MWCNTs	5255 mAh g^−1^/60 mA g^−1^	50 (600 mAh g^−1^/60 mA g^−1^)	2.75/4.31 V	2022	[71]
Holey CNTs	17,500 mAh g^−1^/500 mA g^−1^	150 (500 mAh g^−1^/100 mA g^−1^)	2.75/4.31 V	2022	[72]

**Table 2:** Comparisons of Metal-CO_2_ batteries.

Metal-CO_2_ Battery	Earth’s Crust	Theoretical Potential	Theoretical Energy Density
Li	0.0017 wt%	2.80 V	1876 Wh Kg^−1^
Na	2.3 wt%	2.35 V	1130 Wh Kg^−1^
K	1.5 wt%	2.48 V	921 Wh Kg^−1^

Theoretical energy densities were calculated based on 4M + 3CO_2_→2M_2_CO_3_ + C (M: Li, Na, K).

## Data Availability

Not applicable.
